# Stmol: A component for building interactive molecular visualizations within streamlit web-applications

**DOI:** 10.3389/fmolb.2022.990846

**Published:** 2022-09-23

**Authors:** J.M. Nápoles-Duarte, Avratanu Biswas, Mitchell I. Parker, J.P. Palomares-Baez, M. A. Chávez-Rojo, L. M. Rodríguez-Valdez

**Affiliations:** ^1^ Laboratorio de Química Computacional, Facultad de Ciencias Químicas, Universidad Autónoma de Chihuahua, Nuevo Campus Universitario, Chihuahua, Mexico; ^2^ Doctoral School of Biology, University of Szeged, Szeged, Hungary; ^3^ Biological Research Centre, Szeged, Hungary; ^4^ Molecular and Cell Biology and Genetics (MCBG) Program, Drexel University College of Medicine, Philadelphia, PA, United States; ^5^ Program in Molecular Therapeutics, Fox Chase Cancer Center, Philadelphia, PA, United States

**Keywords:** python package, streamlit, protein visualization, open source, stmol

## Abstract

Streamlit is an open-source Python coding framework for building web-applications or “web-apps” and is now being used by researchers to share large data sets from published studies and other resources. Here we present Stmol, an easy-to-use component for rendering interactive 3D molecular visualizations of protein and ligand structures within Streamlit web-apps. Stmol can render protein and ligand structures with just a few lines of Python code by utilizing popular visualization libraries, currently Py3DMol and Speck. On the user-end, Stmol does not require expertise to interactively navigate. On the developer-end, Stmol can be easily integrated within structural bioinformatic and cheminformatic pipelines to provide a simple means for user-end researchers to advance biological studies and drug discovery efforts. In this paper, we highlight a few examples of how Stmol has already been utilized by scientific communities to share interactive molecular visualizations of protein and ligand structures from known open databases. We hope Stmol will be used by researchers to build additional open-sourced web-apps to benefit current and future generations of scientists.

## 1 Introduction

Scientific web-applications or “web-apps” are powerful tools for sharing computational methods and large datasets exploration with other researchers who may or may not have scientific programming expertise. Creating such web-apps has become increasingly common with many recent studies producing new computational methods for analyzing results from large-scale experimental campaigns, such as high-throughput drug screens or development of machine learning algorithms aiding biomedical sciences. This fundamental breakthroughs can then be easily leveraged by other researchers to make further scientific advancements. However, building user-friendly web-apps can be difficult, since it can be time-consuming and not all researchers possess the front-end coding expertise (e.g., HTML, Javascript, React, Typescript) required to make simple, interactive user interfaces.

Streamlit (https://github.com/streamlit/streamlit) is an open-source library that allows researchers to create web-apps with just a few lines of Python code without any need for front-end coding skills. Already, Streamlit has been extensively used by researchers to make scientific web-apps (Parker et al. (2022); Kui et al. (2022); Absar et al. (2022); Li et al. (2022b); Li et al. (2022a)). These web applications allow an easy and intuitive way that researchers gain novel scientific insights by enabling easy exploration of large data sets. For this, it is easy to deploy basic charts (bar, scatter, etc.) or tabular forms (data frame, tables, etc.). Recently, Lee et al. (2022), published a web-app called “StarGazer,” a hybrid intelligence platform for drug target prioritization and digital drug repositioning using Streamlit front-end, developed by the AstraZeneca team (https://github.com/AstraZeneca/StarGazer).This platform works with multi-source and multi-omics data and is based on a target prioritization scoring system which displays scores for genes related to phenotypic traits in a Streamlit dashboard. Likewise, in the field of biomedical sciences, Kiss et al. (2022) built a web-application for early prediction of acute necrotizing pancreatitis by artificial intelligence, which uses XGBoost machine learning algorithm for the prediction of pancreatic necrosis. The model relies on data from 2,387 patients with acute pancreatitis and was deployed as an online app using Streamlit (http://necro-app.org/). Another recent contribution in the field of computational biology with Streamlit based web-app, is the “iCarboxE-Deep”, a server to identify carboxy-glutamate post-translational modification (PTM) sites Naseer et al. (2022). iCarboxE-Deep web-app, using Deep Convolutional Neural Network (CNN) based classifier can predict the 4-carboxyglutamate sites in protein sequences with increased accuracy and F1-score of 0.874 (https://share.streamlit.io/sheraz-n/carboxyglutamate/app.py). In sum, Streamlit serves as a powerful tool which democratizes the creation of web-apps for sharing scientific software and data.

**FIGURE 1:**
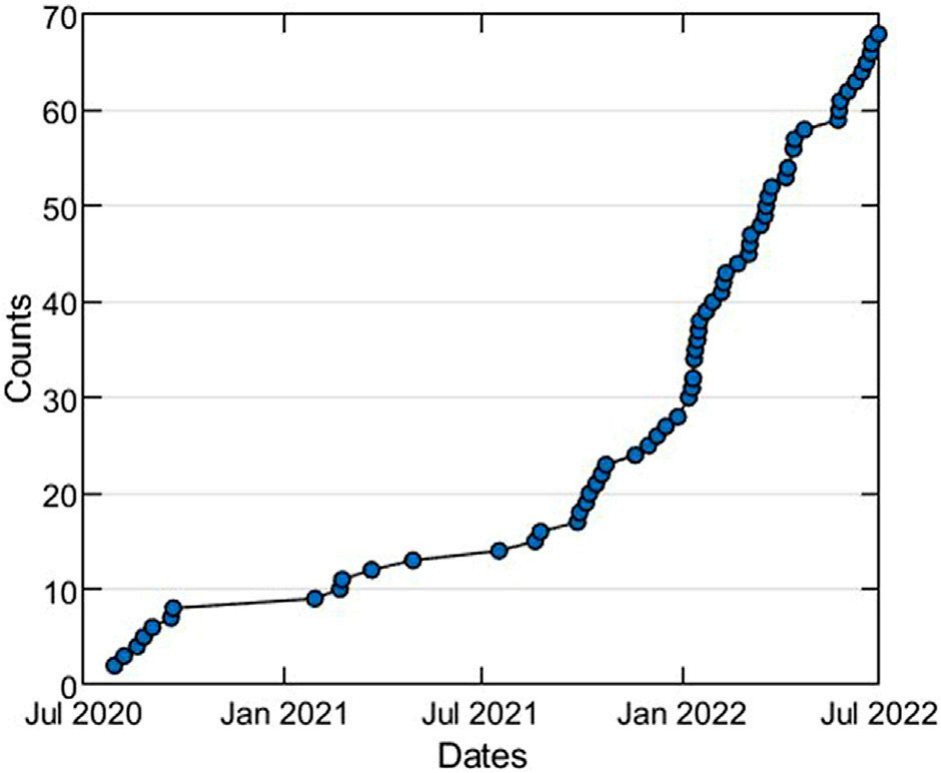
Star history plot of the stmol GitHub repository (*untill July, 2022*).

Structural bioinformatics and cheminformatics have been early adopters of Streamlit for building web-apps to share computational methods and large data sets analysis. However, these fields rely heavily on interactive 3D and 2D molecular visualizations of protein and ligand structures, which is not supported by out-of-the-box Streamlit. Therefore, we created Stmol (https://github.com/napoles-uach/stmol), a component for rendering interactive 3D molecular visualizations of protein and ligand structures within Streamlit web-apps. Stmol utilizes libraries, such as Py3DMol Rego and Koes (2014), and Speck https://github.com/wwwtyro/speck. In Figure 1, we can see the star history for the GitHub repository of stmol project, which shows the constant growing interest of the scientific community towards the component.

In this paper, we demonstrate how to create Stmol rendered molecular visualizations within Streamlit web-apps, introduce how to contribute to the project, and present some use cases of Stmol to give a glimpse how its capabilities.

## 2 Methods

The fundamental aspect of the Stmol package to render 3D molecular visualization at the Streamlit frontend, is highly dependent on the showmol()function. Its value resides in the simplicity that it offers to convert the HTML objects behind py3Dmol. This makes it possible to build a WebGL-based view object structured entirely around Py3Dmol Python package and further calling the Stmol function showmol(), to display a 3D structure in the Streamlit web-app. For instance, an object defined as, obj = py3Dmol.view(query=′pdb:1ubq′), can be easily visualized and interacted at the frontend using, showmol(obj). Albeit, the simplicity of the showmol()function, it offers tremendous extensibility to build more complex visualizations. Furthermore, with the intend to make visualization and handling of 3Dmol/py3DMol objects easier, Stmol package offers several intuitive functions for the user to build web apps using extremely simple Python scripts. Stmol functions can be organized in two broad categories—1) building py3Dmol molecular objects and 2) post-processing molecular objects.

### 2.1 Building py3Dmol objects

Stmol in its current state offers three functions to build py3Dmol objects, each of which returns a py3Dmol object. Functions, with their usage are as follows.

• makeobj—Building the molecular visualization from a file with a valid extension (e.g., pdb,xyz, etc.). Thereby, allowing users to upload and visualize the data
• render_pdb—Quick visualization of structures from the Protein Data Bank (PDB) based on their associated four letter identifier
• obj_upload—Useful to work with Streamlit widget st.file_upload(). Returns the corresponding py3Dmol object from the uploaded file

**FIGURE 2:**
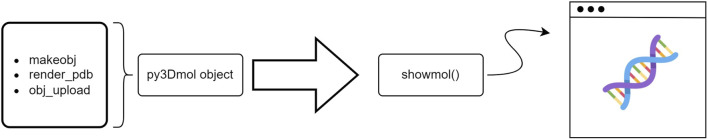
Schematic representation of a typical workflow to create py3Dmol objects and visualize using Stmol package at the user-end.

Each of the above functions, can be easily implemented for building py3Dmol objects and visualized using the showmol() function as shown in Figure 2.

### 2.2 Post-processing py3Dmol objects

Likewise, the py3Dmol objects can be further customized. In the context of making Stmol, a tool to leverage this processes at ease, we added few post-processing functions which are enlisted below:

• add_hover—Viewing atom’s labels on mouse hovering
• render_pdb_resn,render_pdb_resi—Labelling residues within a protein
• add_box,add_sphere,add_cylinder—Adding basic geometries
• add_model—Aggregate a new model to an existing py3Dmol object

**FIGURE 3:**
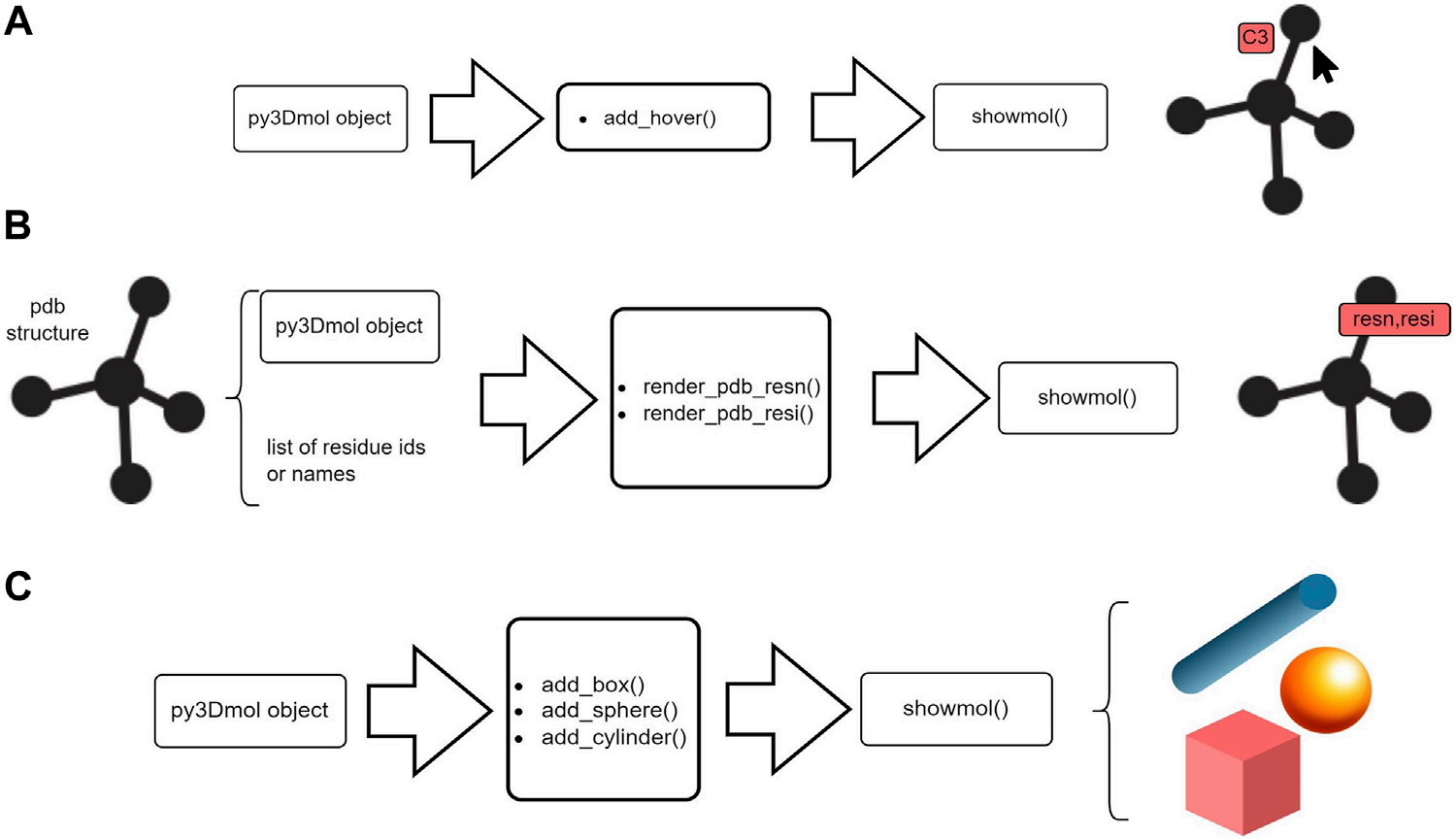
Schematic representation of post-processing utilities offered by Stmol. **(A)** Hover function to obtain atom’s information. **(B)** Rendering residue to add residue labels. **(C)** Adding simple geometries.

Figure 3, demonstrates the flow of work for some of these functions. These functions add new features to existing py3Dmol objects.

The Stmol package aims to broaden the molecule rendering functionalities. We are open for contributions to the Stmol project repository in GitHub (https://github.com/napoles-uach/stmol). Two possible ways to contribute to the stmol library are, with the static HTML components and with the bidirectional components, the latter which sends and receives information to/from the backend. The tree of the directories is shown below:

stmol/
 __init__.py
 utils.py
 example.py
 front-end/
  public/
  src/
   MyComponent.tsx

The relevant folders for the purpose of making contributions are included in the tree.

Here we briefly explain how to proceed if new features are going to be contributed to the project using simple examples.

### 2.3 Static contributions

A recent addition in a form of static HTML wrapper is the speck_plot function, which helps in rendering Speck structures (https://github.com/wwwtyro/speck) within the Streamlit web-application. In brief, Speck is an open-source browser based WebGL molecule renderer written primarily in the JavaScript programming language, that produce attractive high quality molecular figures displaying salient features such as ambient occlusion, pixel-perfect atoms and bonds, depth-aware outlines, and depth of field. The speck_plot static wrapper incorporates the Python packages such as ipyspeck and ipywidgets. An HTML string is passed at the front-end using Streamlit’s components.html() API.

# Modules
import streamlit.components.v1 as components
import ipywidgets as widgets
from ipywidgets import embed
import ipyspeck
# Static wrapper function
def speck_plot(_xyz, wbox_height="700px",
 wbox_width="800px”, component_h = 700,
 component_w = 800, scroll = False):
# Read the xyz file
spec_xyz = ipyspeck.speck.Speck(data = _xyz)
# Create the widget box
widg = widgets.Box([spec_xyz],
layout=widgets.Layout(height=wbox_height,width=wbox_width))
# Embed the widget box in the streamlit html component
sc = embed.embed_snippet(widg)
html = embed.html_template.format(title="", snippet=sc)
components.html(html,height =
component_h, width = component_w,scrolling=scroll)
return spec_xyz

**FIGURE 4:**
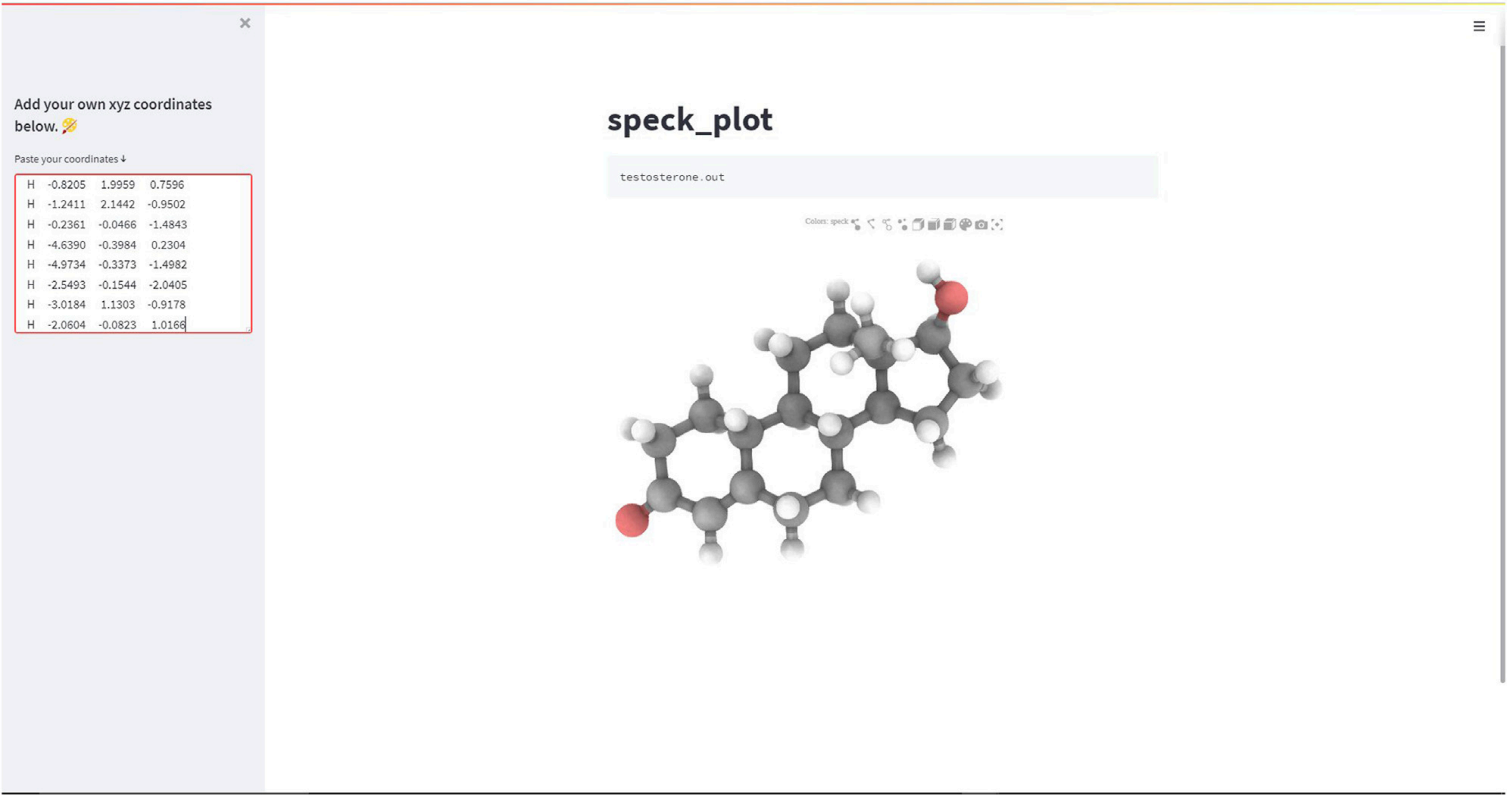
Screen-capture of the Streamlit web-app demonstrating the speck-plot rendering function.

Thus, importing the speck_plot function from the stmol module along with a few simple Streamlit syntaxes can be used to build and deploy a minimalist Streamlit web-app (Figure 4).

### 2.4 Bidirectional contributions

The Bidirectional component inclusion, requires implementation of both back and front-end–commonly written using HTML, Javascript, React, Typescript, etc. A bidirectional Component in Streamlit has two parts, the front-end and a python API. The frontend, is build in HTML, plus React or Typescript code, and it is possible to incorporate other packages using the npm package manager. On the other hand, the Python API is used to instantiate and communicate to the front end. Stmol currently does not have this kind of functionality which could have some advantages like retrieving the atoms or ligands information by clicking on the atoms. We are still exploring this possibility, and it is part of the plan for Stmol in the near future. Further information on bidirectional components can be found in the Streamlit documentation (https://docs.streamlit.io/library/components).

Currently, there are a few known limitations of Stmol. First, Stmol does not provide in-built highlighting option for the protein structure (e.g., drug binding sites). Second, the present static Stmol wrapper lacks bidirectional functionality. Third, the speck_plot function is currently only compatible with. xyz format of proteins and lacks argument to tweak with the visualized protein structure. Therefore, we plan to include such functionalities in future releases of Stmol. Further, we call the community to participate and contribute to the improvement of this open-source project.

## 3 Results

As discussed previously, the development of any web-app with Streamlit front-end, requires only the knowledge of Python programming. In this section, we will be implementing the stmol Streamlit component and build a demo Streamlit web-app. Stmol depends on following Python libraries, streamlit, py3Dmol, ipyspeck and ipywidgets which are installed together with the package. The pip python package manager can be used for the installation of the mentioned packages with the following command,

pip install stmol

The root folder consists of the python file app.py (refer to the *GitHub folder*) consisting of following few lines of code.

#Importing the installed libraries
import streamlit as st
from stmol import showmol
import py3Dmol
#1.Calling Streamlit widgets
st.sidebar.title(′Demo app′)
style = st.sidebar.selectbox(′style′, [′cartoon′, ′stick′, ′sphere′])
#2.Using Py3Dmol methods
xyzview = py3Dmol.view(query=′pdb:1A2C′)
xyzview.setStyle({style:{′color′:′spectrum′}})
xyzview.setBackgroundColor(′white′)
#3.Calling the stmol function called showmol
showmol(xyzview, height = 500,width=800)

The above code is almost self-explanatory. The sections to emphasize can be well divided into:

1) Calling Streamlit widgets
2) Using Py3Dmol methods
3) Calling the stmol function called showmol to render the resulting protein

The app.py file, can run locally from the command terminal with the following command,

streamlit run app.py

**FIGURE 5:**
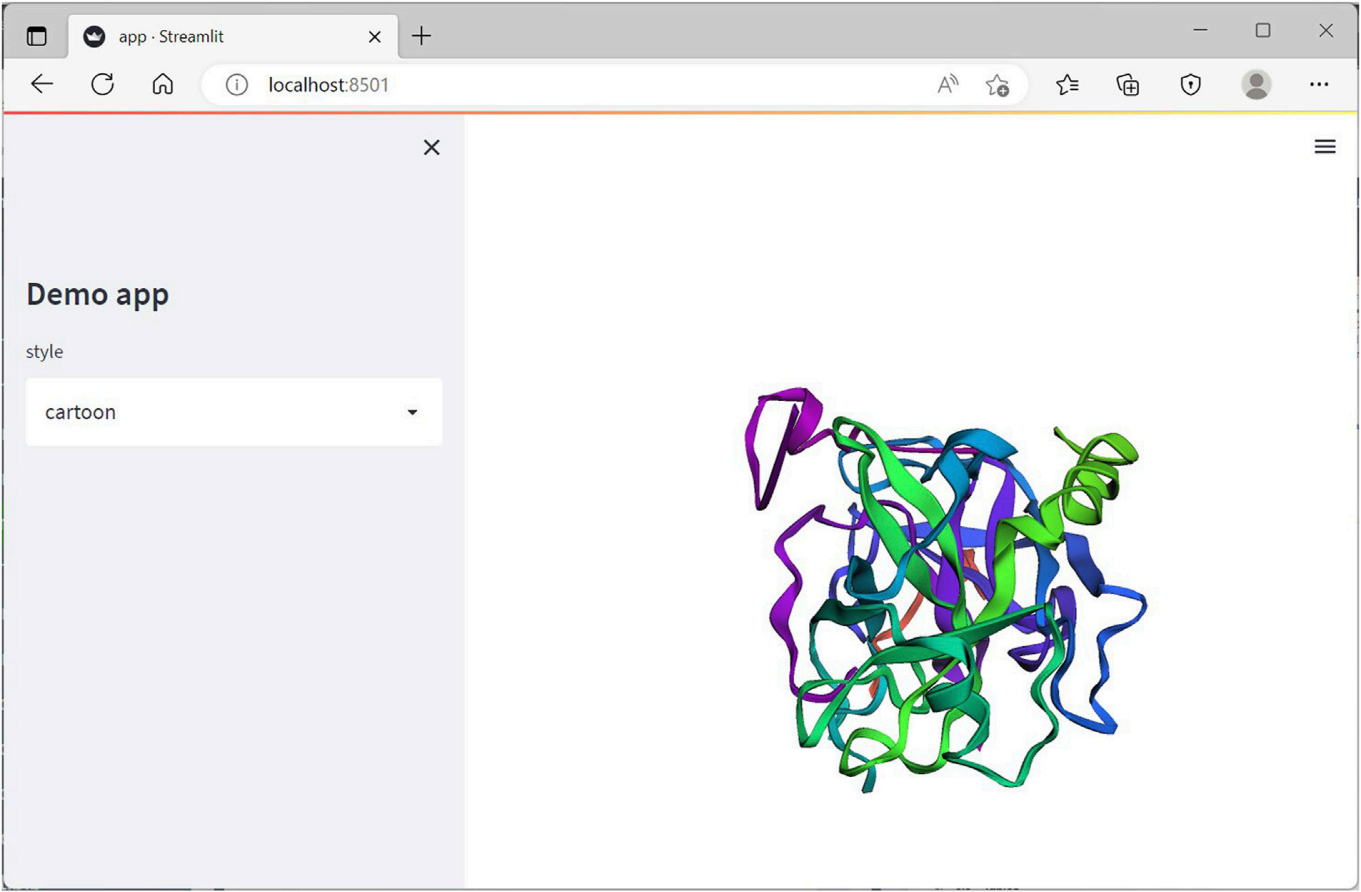
Streamlit web-app running on the browser on local server on executing the app.py Python file.

The resulting web-app on running successfully should appear in a new tab of the default internet browser window as a localhost (Figure 5).

**FIGURE 6:**
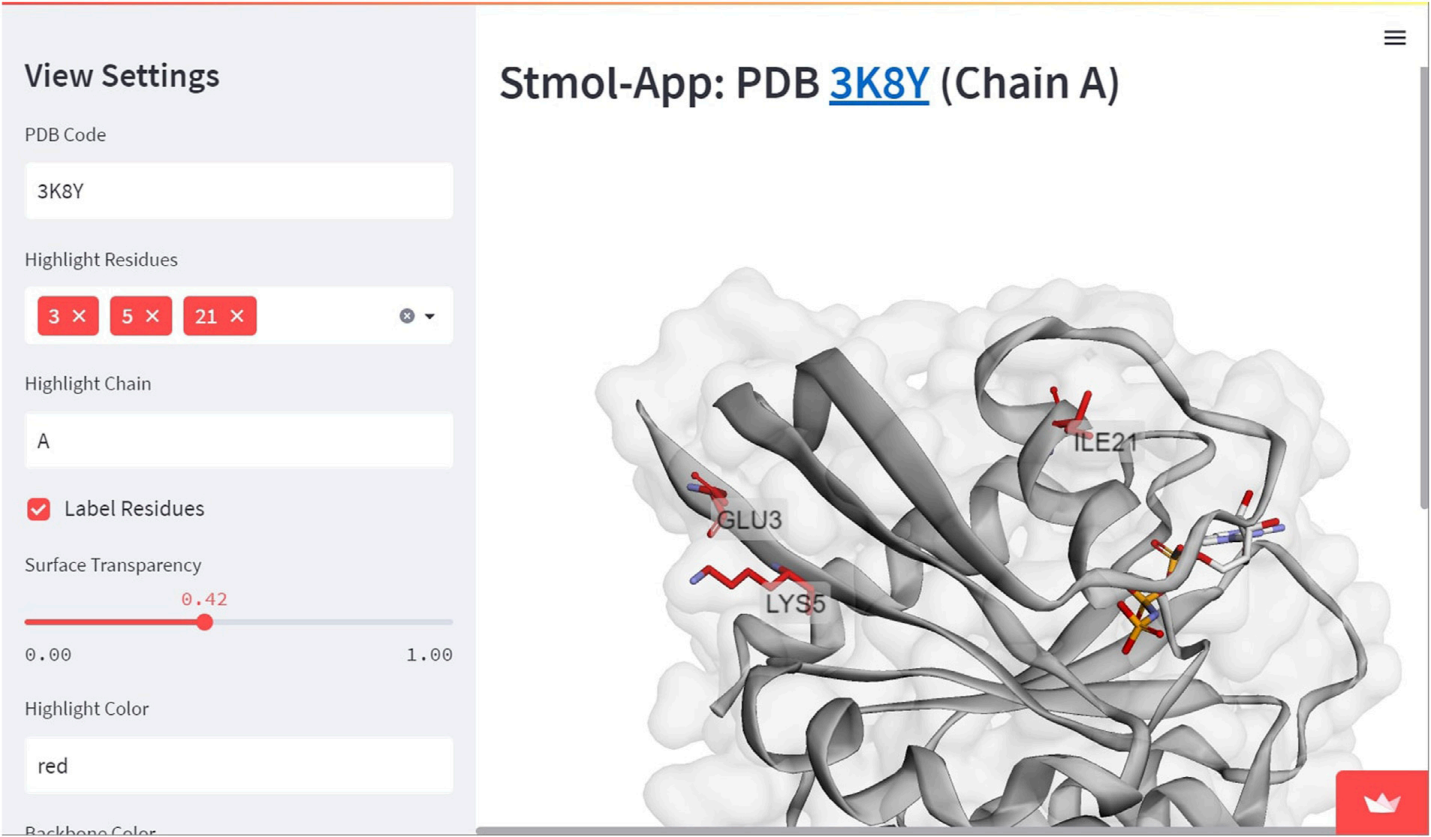
Demo application. This is a more advanced example where residues are highlighted and molecular surface is included with transparency. In this case, the user experience is enriched with Streamlit widgets.

In order to deploy the web-app for external usage and global availability over internet, a requirements.txt file needs to be created, which consists of the dependencies crucial for running the web application. The common practice in creating requirements.txt file is by using Python libraries, such as pipreqs. In the above demo app, the content of the requirements.txt file consists of streamlit, stmol and py3Dmol. The application can be hosted over Streamlit Cloud (https://streamlit.io/cloud), which utilizes code pushed to a Github repository. With future Streamlit Cloud pulls, any changes in the code alteration from the GitHub path will be automatically (or can be manually) rebooted. Demo application and examples can be accessed through this link (See Figure 6 for a Screenshoot).

### 3.1 Use cases of STMOL

In this section we aim to briefly highlight few significant implementations of Stmol package in development of scientific web-apps.

**FIGURE 7:**
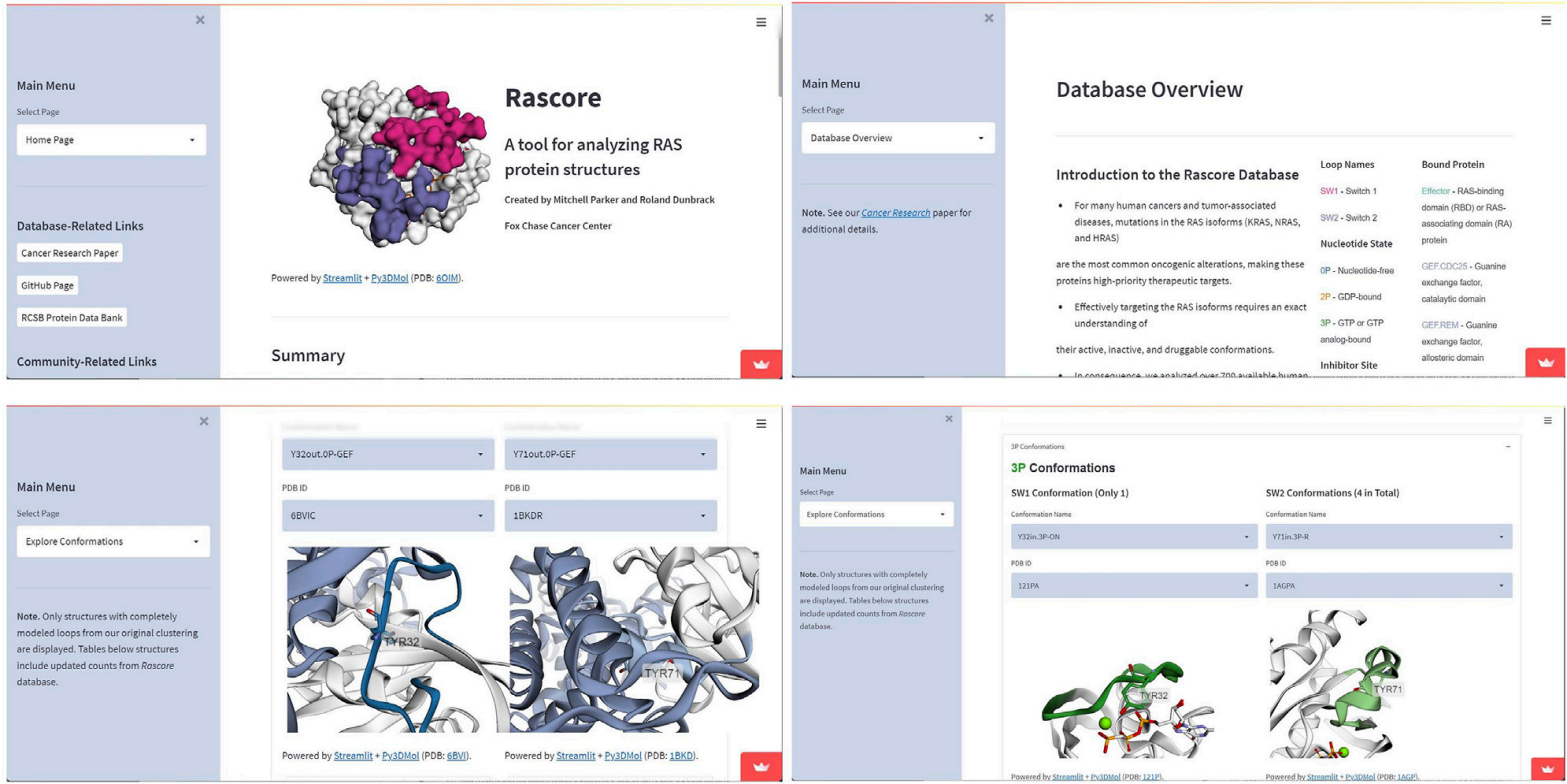
Screen-captures showing use case of the Stmol package for the development of the Rascore Web-app. Among its options are “Main Menu”, “Database Overview” and “Explore Conformation” sections where Stmol is used to visualize the structures.

**Example 1—Rascore.** Rascore (Parker et al., 2022) is a Streamlit based web-app for analyzing the 3D structure of the tumor-associated RAS proteins (KRAS, NRAS, and HRAS—the most common cancer drivers). Rascore allows researchers to quickly benchmark candidate RAS inhibitors (through structure-based and ligand-based approaches) with other RAS directed inhibitors previously tested, providing a data-driven approach for accelerating RAS drug discovery. Rascore helps scientists to explore and compare published structural models of RAS proteins in the Protein Data Bank (PDB) in ways that simplify biological study of these proteins and facilitate RAS drug discovery. The code can be found at https://github.com/mitch-parker/rascore. The Rascore server utilizes py3Dmol for residue annotation and finally renders the molecular object to the Streamlit frontend using the showmol() function, shown in Figure 7. In the latest release of Stmol, render_pdb_resn() function can be used for the same purpose. Implementation of this similar feature has been added as an example application here—https://napoles-uach-stmol-home-pom051.streamlitapp.com/Examples.

**FIGURE 8:**
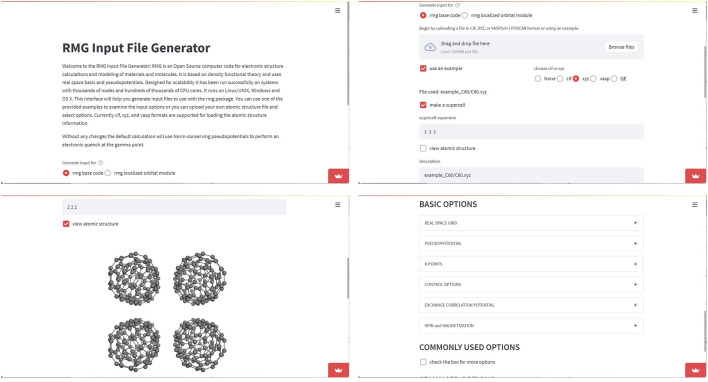
Screen-captures of RMG web-app. The *showmol*() function was called for the purpose of rendering crystalline structures. The end user can select the options accepted by the RMG code and visualize the crystal using repetition cells.

**Example 2**—**RMG web based input file generator.** RMG Input File Generator is an user interface developed by the creators of RMG Briggs et al. (1996), a Density Functional Theory code for electronic structure calculations for the modeling of materials and molecules. It allows to build the input files, by following provided examples or by upload of atomic structure files, shown in Figure 8. The code can be found at https://github.com/RMGDFT/rmgwebinterface. The molecular rendering of the *.xyz* file format can be further simplified using the obj_upload() function of Stmol package. This example makes evident that Stmol is being also used in the field of Computational Materials Science, where visualization of 3D structures is also of importance.

**FIGURE 9:**
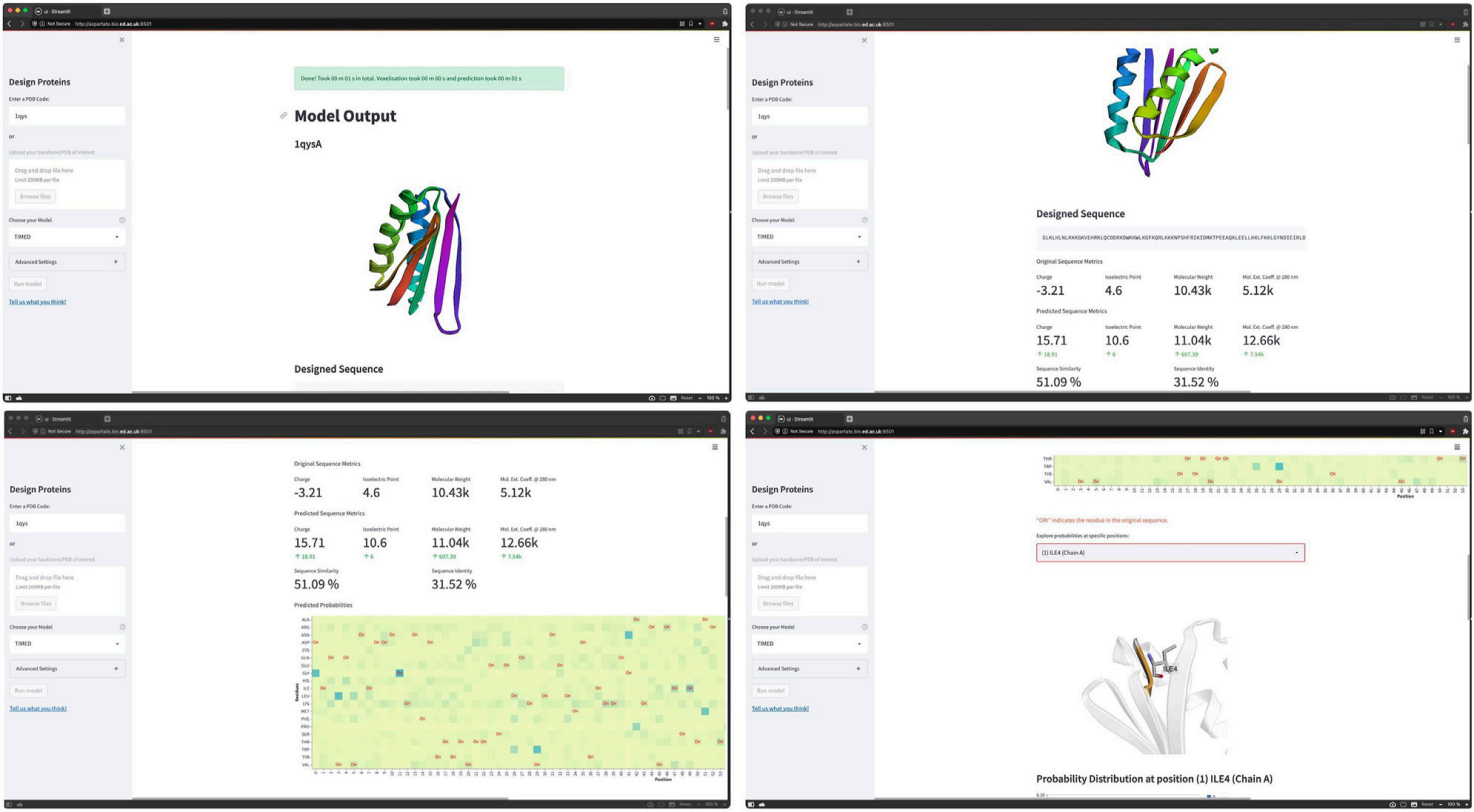
Screen-captures of TIMED web app. Images courtesy of Leonardo V. Castorina who is one of the contributors of the TIMED project. TIMED tackle the “Inverse Folding Problem” aiming to identify the residue sequence that will reliably fold into a given structure.

**Example 3**—**TIMED user interface.** TIMED (Three-dimensional Inference Method for Efficient Design) is a Convolutional Neural Network model trained to solve the “Inverse Folding Problem” (Castorina et al. (2022)). The input for TIMED is a 3D shape (empty backbone) and the output are the subunits at the positions of the backbone. The Streamlit user interface for TIMED allows to select a given protein by the PDB code or uploading a backbone/PDB file and run the model to get probabilities at specific positions. The code can be accessed at https://github.com/wells-wood-research/timed-design, shown in Figure 9.

## 4 Discussion

We present an open-source python package, stmol, which serves as a plugin to render 3D molecular visualization of protein within Streamlit web-apps. The Stmol component enables researchers from different scientific backgrounds with varying front-end programming expertise to quickly and easily deploy interactive web applications to serve several purposes, such as visualization of experimental or simulated data among several scientific communities working on similar projects. This tool has been well embraced by the science enthusiasts within the Streamlit community, as evident from the constant growth of users since its first release.

Stmol provides an easy way for bioinformatic and cheminformatic researchers to create web-apps that guide structure-based and ligand-based drug design through molecular visualizations. In this paper, we highlighted Rascore (Figure 7) a Streamlit web-app which utilizes Stmol for visualization of drug-bound (and unbound) structures of a high-priority cancer target called RAS proteins. Since the launch of Rascore in April, this web-app has accrued over 3,000 users from academia and the pharmaceutical industry who are using the app to guide development of the next generation of RAS inhibitors. Similarly, we envision other researchers will use Stmol to quickly build Streamlit web-apps for the purpose of streamlining drug discovery related to other therapeutic targets. In other scenarios, due to its nature, the contribution of Stmol to drug discovery is limited to the task of visualization, and in this sense it has the potential to be used in conjunction with other packages (e.g., Rdkit, ProLIF, Vina) to build molecular docking interfaces on the browser with all the benefits that this implies, being the reduction of costs in specialized software one of them. We envision other possible applications, but some will require improvements in the library that are for now in the future plans for this project, like improving the interactivity and retrieval of information e.g. displaying atom distances, and also features more oriented to drug discovery.

Although there are many possible improvements to Stmol, we believe that this is a project that deserves attention, and that its use along with Streamlit will be more common in future publications by the bioinformatics community. Nowadays, for Stmol the main objective is to make it simpler for scientist to get through the process related to visualization of molecular structures in the deployment of web apps with Streamlit.

## Data Availability

Publicly available datasets were analyzed in this study. This data can be found here: https://github.com/napoles-uach/stmol.
